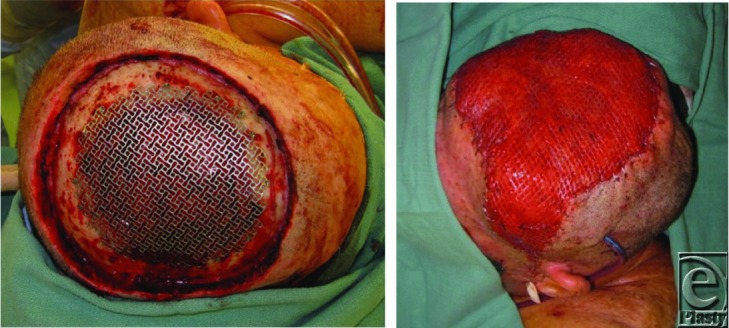# Scalp Reconstruction With Free Latissimus Dorsi Muscle

**Published:** 2013-07-19

**Authors:** Danielle H. Rochlin, Justin M. Broyles, Justin M. Sacks

**Affiliations:** Department of Plastic and Reconstructive Surgery, The Johns Hopkins University School of Medicine, Baltimore, Md

**Keywords:** Scalp reconstruction, Head and neck reconstruction, Calvarium Reconstruction

**Figure:**
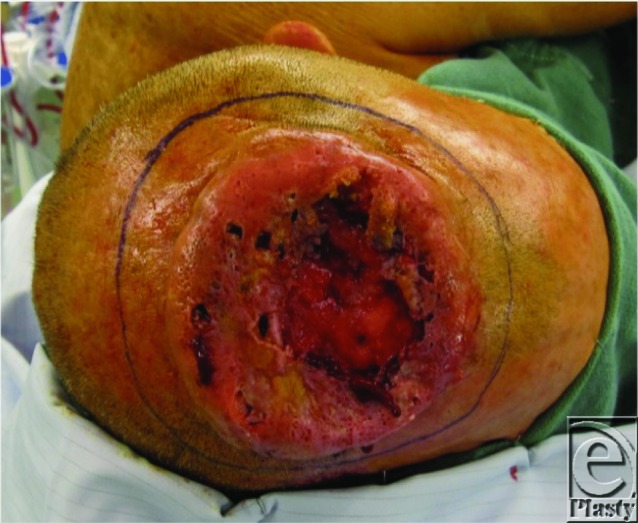


## DESCRIPTION

A 61-year-old man presents with invasive basal cell carcinoma of the scalp. Composite resection results in a 280-cm^2^ scalp defect of the outer and inner table. Titanium mesh was used to reconstruct the calvarial defect, while reconstruction of the scalp wound was accomplished with a free latissimus dorsi (LD) flap plus split-thickness skin graft.

## QUESTIONS

1. **What are the advantages and indications for using a free LD flap in scalp reconstruction?**
2. **What are the expected outcomes and complications?**
3. **Are there any inherent challenges or limitations in working with the free LD flap?**
4. **What steps should be taken in the preoperative planning phase?**

## DISCUSSION

The scalp consists of 5 anatomical layers, conveniently remembered with the mnemonic scalp: skin, subcutaneous tissue, galea aponeurosis, loose areolar tissue, and pericranium.^1^ The galea borders the frontalis and occipitalis muscles and extends laterally as the temporoparietal fascia. Characteristic properties of scalp tissue include its thickness (3-8 mm), inelasticity due to the galea and pericranium, and hair-bearing quality. While its thickness makes the scalp a favorable donor site for split-thickness skin grafting, the latter 2 properties complicate primary closure and make reconstruction esthetically challenging, respectively. Scalp defects may violate any number of scalp layers and can occur secondary to burns, trauma, chronic postsurgical infection or scarring, and tumor extirpation. Scalp neoplasms, in particular, pose several reconstructive challenges due to the need for wide excision that may include the calvarium, the potential for disease recurrence, and radiation-induced tissue necrosis.^2^

Reconstructive options vary on the basis of the location and size of the defect, and careful preoperative characterization of the wound is essential for successful reconstruction.^1^^,^^3^ Partial-thickness wounds can heal by secondary intention and may benefit from split-thickness skin grafting if there is intact pericranium or fascia to serve as a vascularized wound bed, particularly if the defect is large or in a highly visible area. For full-thickness wounds, primary closure is sufficient for small defects (<2 cm^2^), though wide undermining and scoring of the galea is often necessary due to limited tissue elasticity. Local flaps may also be utilized if primary closure is not possible. Moderate defects (2-25 cm^2^) can be reconstructed with local tissue rearrangement such as rotation flaps with skin grafting of the donor site. Large defects (>25 cm^2^) may require multiple or larger rotation flaps, tissue expansion, or free tissue transfer (FTT). Tissue expansion enables replacement with local hair-bearing skin, yet this option is not feasible if there is limited scalp tissue or if expediency is a priority, as in the case of malignancy, because expansion requires staged procedures with a long interval period. Free tissue transfer allows for a single-stage reconstruction and is also favored in cases involving infected calvarium or irradiated tissue.^2^ Free tissue transfer–based scalp reconstruction with an omental flap was first described by McLean and Buncke in 1972 and has since been accomplished with the LD muscle flap, anterolateral thigh flap, radial forearm flap, rectus abdominus muscle flap, scapular flap, and serratus muscle flap.^4^

The LD muscle flap is one of the most popular flaps for scalp reconstruction due to the large size of the LD muscle and potential for broad coverage. The flap can be combined with a skin graft to give a pleasing esthetic and has long and reliable vascular pedicle. Anastomoses are preferentially made with superficial temporal or occipital vessels, with use of vessels in the neck if necessary.^2^ Preoperatively, it is critical to assess the recipient vessels. For central scalp reconstructions, vein grafts need to be considered if the temporal or occipital vessels are unavailable. Unlike composite flaps, which often require secondary debulking procedures, free muscle flaps atrophy and contour favorably to the skull over time.^1^ Though thinning and atrophy risk recurrent skull and hardware exposure, a comparison of 100 free muscle flaps with split-thickness skin grafts (81 LD flaps) to 38 fasciocutaneous or myocutaneous flaps (3 LD flaps) demonstrated equal reliability between the 2 reconstructive options.^5^

Outcomes following LD flap-based scalp reconstruction are favorable with rare instances of graft loss and minimal complications. In the aforementioned study of 138 free flaps (84 LD flaps), total flap loss occurred in 3 patients (2%) with 1 loss involving an LD flap that occurred secondary to arterial thrombosis.^5^ In a separate retrospective study, scalp reconstruction with 37 free flaps (16 LD flaps) following tumor invasion led to successful outcomes in all patients, despite the advanced state of most scalp tumors.^2^ An additional study comparing 16 LD flaps with 17 anterolateral thigh flaps for reconstruction of scalp defects reported equal efficacy of the 2 flaps, without any total LD flap loss.^4^ For the LD flap group, the rates of major (eg, total flap loss, hematoma, and major infection) and minor (eg, partial flap loss, wound breakdown, and minor infection managed with antibiotics) complications were 6% and 31%, respectively. Risk factors for complications included radiation, chemotherapy, larger defect size, smoking, hypertension, diabetes mellitus, and younger patient age likely related to more aggressive resection. One of the most well-known complications of the LD flap is donor site seroma, with a reported rate of formation ranging from 9% to 80% depending on the dissection and fixation technique.^6^ Though typically regarded as a minor complication, seromas may lead to wound infection and dehiscence without appropriate management.

Cases involving tumor invasion of the skull require partial- or full-thickness removal of the calvarium in addition to cranioplasty and soft tissue reconstruction. When scalp tissue is infected, calvarial reconstruction should be delayed pending successful debridement and antibiotic therapy. Repair of cranial defects may be accomplished with autologous bone grafts, such as split calvarium or rib, or alloplastic materials such as titanium mesh or methyl methacrylate.^7^ Autologous grafts are associated with less graft loss and may allow for management of infection without complete graft removal. Titanium mesh can cover sizable defects without donor site morbidity, though infection occurs more frequently compared to bone grafts.^7^ Methyl methacrylate is also regarded as a durable prosthetic material with favorable contouring properties, yet the hardening reaction is exothermic and requires cool irrigation to prevent thermal damage. Regardless of the reconstructive material, simultaneous calvarial and scalp reconstruction has been shown to be feasible without a significant increase in complications.^2^^,^^4^^-^5 New computer-assisted imaging techniques may also be utilized in the preoperative planning phase to model bony defects and optimize functional and esthetic outcomes of reconstruction.^8^

**Figure:**